# Gastrointestinal Pathogens in Multi-Infected Individuals: A Cluster Analysis of Interaction

**DOI:** 10.3390/microorganisms11112642

**Published:** 2023-10-27

**Authors:** Joy Backhaus, Hagen Frickmann, Ralf Matthias Hagen, Gustavo Concha, Ernst Molitor, Achim Hoerauf, Simone Kann

**Affiliations:** 1 Statistical Consulting, 97074 Wuerzburg, Germany; statistik.backhaus@gmail.com; 2 Department of Microbiology and Hospital Hygiene, Bundeswehr Hospital Hamburg, 20359 Hamburg, Germany; frickmann@bnitm.de; 3 Department of Medical Microbiology, Virology and Hygiene, University Medicine Rostock, 18057 Rostock, Germany; 4 Department of Microbiology and Hospital Hygiene, Bundeswehr Central Hospital Koblenz, 56070 Koblenz, Germany; ralfmatthiashagen@bundeswehr.org; 5 Organization Wiwa Yugumaiun Bunkauanarrua Tayrona (OWYBT), Department Health Advocacy, Valledupar 2000001, Colombia; gustavoconcha16@gmail.com; 6 Institute of Medical Microbiology, Immunology and Parasitology (IMMIP), University Hospital Bonn, 53127 Bonn, Germany; molitor@uni-bonn.de (E.M.); achim.hoerauf@ukbonn.de (A.H.)

**Keywords:** *Blastocystis hominis*, *Dientamoeba fragilis*, *Giardia lamblia*, gastrointestinal infections in indigenous, interactions, mathematical estimations

## Abstract

Indigenous people live in remote areas of Colombia. Multiple infections with bacteria, protozoa and/or helminths are common, as well as colonization in various forms. This study focused on the question of whether and to what extent various pathogens interact with each other. Therefore, a mathematical approach was retrospectively applied to PCR-based data of 244 stool samples, collected in two datasets. A stable cluster solution of the pathogens assessed was determined, and a unique configuration between *Blastocystis hominis*/*Campylobacter* spp./*Giardia lamblia* forming cluster 1 and *Dientaemoeba fragilis* was verified. A pathogen density-dependent interplay appeared between the *B. hominis*/*Campylobacter* spp./*G. lamblia* cluster, *D. fragilis* and *Ascaris lumbricoides*. The applied mathematical approach demonstrated that co-infections with parasites of questionable pathological relevance such as *B. hominis* and *D. fragilis* can be of diagnostic relevance due to their ability to promote or repress other pathogens. With the increasing availability of highly sensitive multiplexed molecular diagnostic approaches even in resource-limited settings, where multiple colonization of infection events with enteric pathogens in parallel are common, the importance of interpreting whole pathogen patterns rather than just individual pathogen detection may become more and more relevant.

## 1. Introduction

The Indigenous tribes called Wiwa and Kogui live in retracted areas of the Sierra Nevada de Santa Marta, in the north-east of Colombia. Their living conditions are very difficult, e.g., with regard to socio-economic circumstances. High gastrointestinal infection rates are common, as they fetch water from rivers and/or unprotected wells. Further, they perform traditional agriculture (top dressing) and live in simple clay huts with palm roofs. While the livestock from the Koguis are kept in fences near their houses, the livestock from the Wiwas live mainly within their houses. Education is sparse, and aid programs are rare. Medical care for the regions is sparse. Many inhabitants have to walk about six hours to reach the nearest health point, which is poorly equipped. For most patients, the next hospital is another six hours away. Although there are health brigade programs to improve the situation, they focus mainly on preventive medicine (e.g., vaccinations) and stay only for a maximum of 2 days in a zone. Additionally, they perform symptomatic treatment only, and causes of diseases are hardly investigated at all. This is one component of the multifactorial circumstances which result in a high gastrointestinal pathogen burden and enteric dysbiosis found by our group in 2014 [1,2], as well as in subsequent analyses [3,4,5,6]. Hereby, stool samples were analyzed with PCR techniques for microsporidia (*Encephalitozoon* spp.), helminths (*Strongyloides stercoralis*, *Hymenolepis* spp., *Ascaris lumbricoides*, *Taenia* spp., *Trichuris trichiura* and *Necator americanus*), protozoa (*Blastocystis hominis*, *Giardia lamblia*, *Dientamoeba fragilis*, *Entamoeba histolytica* and *Cryptosporidium* spp.) and bacteria (*Shigella* spp./enteroinvasive *Escherichia coli* (EIEC), *Campylobacter* spp., *Aeromonas* spp. and *Salmonella* spp.).

Multiple infections were found in nearly all volunteers, whereas the etiological relevance of individual positive test results as well as the potential interaction of pathogens could not be statistically scrutinized [1,2,3,4,5,6]. Based on two independent datasets of more than 100 Indigenous individuals, from the previous assessments we aimed to analyze whether the pathogens may be intertwined. The three mayor aims of the study were to figure out if (1) different pathogens act in a synergistic/protective or (2) in an inhibitory/non-protective way, and (3) whether there are pathogen groups that favor each other.

## 2. Materials and Methods

### 2.1. Ethical Approval

This study was conducted in line with the Declaration of Helsinki. Its protocol was approved by the Ethics Committee of Valledupar, Cesar, Colombia (Act. no 0022013). Written informed consent was obtained from each participant as well as from a parent or legal guardian of a child before participation. All participants were informed about their results and received treatment, if appropriate.

### 2.2. Laboratory Techniques

Laboratory assessment was performed as described in detail elsewhere [5,6]. Shortly summarized, the stool specimens were stored in a cooling box for transportation and deep-frozen at −20 °C until the day of shipment to Germany. Shipment was performed in line with national and international regulations by World Courier (Frankfurt, Germany) on dry ice. Prior to the assessments, sample storage was performed at −80 °C, and then nucleic acid extracts were prepared and stored at the same temperature. Nucleic acid extraction was conducted applying the automated Nimbus extractor (See-Gene, Seoul, Republic of Korea) in line with the manufacturer’s protocol. Subsequently, the real-time PCR kits Allplex GI-Bacteria (I), Allplex GI-Parasite and Allplex GI-Helminth (I) (SeeGene, Seoul, Republic of Korea) were used on a CFX96 qPCR machine (Bio-Rad Laboratories, Inc., Hercules, CA, USA) and analyzed by applying the the latest SeeGene Viewer software, according to the manufacturer’s instructions within the CE-ICD label in a laboratory accredited according to DIN EN ISO 15189. Details on the diagnostic accuracy of those assays can be found elsewhere [7,8]. In summary, the kits are able to detect microsporidia as well as a variety of helminths (*Encephalitozoon* spp., *Strongyloides stercoralis*, *Hymenolepis nana*, *Ascaris lumbricoides*, *Taenia* spp., *Trichuris trichiura* and *Necator americanus*), protozoa (*Blastocystis hominis*, *Giardia lamblia*, *Dientamoeba fragilis*, *Entamoeba histolytica* and *Cryptosporidium* spp.) and bacteria (*Shigella* spp./enteroinvasive *Escherichia coli* (EIEC) (without further discrimination of the two of them), *Campylobacter* spp., *Aeromonas* spp. and *Salmonella* spp.).

Cycle threshold (CT)-value-based semi-quantification is automatically provided by the software of the real-time PCR device. CT cutoff values, as defined by the assays’ manufacturer, are in the 43 to 45 range, and amplification curves are automatically assessed by a software algorithm to exclude fluorescence artifacts.

Based on our experience with evaluation studies including well-characterized in-house PCR assays as reference standard [7,8,9], the technical detection limit range is within the range of 10^2^ to 10^3^ copies/µL.

### 2.3. Statistical Methods

Statistical analyses were carried out using the R 3.6.1 [10] packages dplyr 2.3.0, fpc2.2-10, mclust 6.0.0, vegan2.6-4, interactions 1.1.5 and ggplot2 3.4.2. The focus of the analyses was the detection of non-random patterns between pathogen combinations. By utilizing CT values of pathogens, data became metric, and this allowed a much more sophisticated approach for data analysis. In a prior publication [1], a binary data approach was used, resembling whether a parasite had occurred or not. Binary data, however, limit available algorithms for estimation, in particular for multivariate statistical approaches.

### 2.4. Descriptive Analysis

Descriptive information includes mean (*M*), standard deviation (*SD*), and number of occurrences (n). In the case of a binary predictor, a Welch test [11] was computed; otherwise, an ANOVA [12] was used. Of note, within these statistical approaches, tests for univariate normality are regarded not essential [12].

### 2.5. Inferential Analysis

Cluster analysis was used to identify non-random patterns in dataset I. This statistical approach is considered a data mining technique, which allows incorporating multiple variables at once in order to characterize their interplay on hierarchical levels. We used k-means hierarchical clustering to inspect whether hierarchical relationships between pathogens can be detected. Ward 2 was employed as a measure for similarity/dissimilarity. Competing methods were used to ensure rival model testing in cluster analysis by applying bootstrapping. We used 10.000 bootstrap resamples. The Jaccard index was used as an indicator of cluster stability [13]. An average Jaccard index <0.6 indicates a rather unstable solution, an average Jaccard index between 0.60 and 75 indicates that a systematic data pattern has been determined, and an average Jaccard index >0.85 indicates a highly stable solution. Once a stable solution was determined by the aforementioned techniques, we proceeded to employ the same approach to dataset II. In the case that the interaction of clusters required scrutinizing, moderated regression was employed. Given that CT values are considered continuous, the moderator was segmented in *SD*.

### 2.6. Datasets

We employed two datasets from different populations to scrutinize the interplay of pathogens by utilizing cluster analysis. At first, we described the medical characteristics of the two independent datasets, and then proceeded to employ the cluster analysis. Cluster analysis was employed exploratorily to dataset I, whereas dataset II served the purpose of validation or falsification.

Dataset I

Dataset I consisted of a total of 150 stool samples from the Kogui people, comprising 12 samples from Avingüe, 21 from Chenducua, 12 from Mamangueka, 66 from Marwamake, 7 from Pueblo Hernández and 32 from Sarachui. The average age of the Koguis was 23.4 years (*SD* = 18.6). In total, 23 samples were from children aged 0–6 years, 61 from adolescents aged 6–18 years and 66 from adults older than 18 years.

Dataset II

Dataset II consisted of a total of 95 stool samples from Wiwa individuals. Thereby, 4 came from Ashintukwa, 12 from Siminke, 39 from Tezhumke and 39 from Cherua. The average age was 21.5 years (*SD* = 15.5). Of the total, 3 samples were provided by children aged 0–6 years, 56 from adolescents aged 6–18 years and 35 from adults older than 18 years.

## 3. Results

### Differences between Datasets

Datasets differed regarding region of origin and Indigenous tribes; differences regarding age, however, were not statistically significant. The number of individuals infected with at least one bacterium or protozoon differed significantly (Table 1).

Multiple infections, as indicated by the maximum number of infections per person, occurred significantly more frequently in Kogui individuals.

Whereas individuals infected with helminths did not differ significantly over the datasets, the number of (different) helminths was significantly higher for dataset I. For bacteria and protozoa, significant differences for the total number of detected infections occurred as well.

Neither for dataset I (*F*(2147) = 2.63, *p* = 0.075) nor dataset II (*F*(2,91) = 0.58 *p* = 0.558) were differences regarding the distribution of helminth infections between age groups found. For protozoa, we found that adults (*M* = 1.49, *SD* = 0.74) had significantly less infections than adolescents (*M* = 1.95, *SD* = 0.67, p_adj_ = 0.008) in dataset II. Children ranged in between (*M* = 1.67, *SD* = 0.57). Regarding bacteria, significant differences were found between the age groups in dataset I, with adults (*M* = 0.394, *SD* = 0.58) showing the lowest number of infections when compared to adolescents (*M* = 0.803, *SD* = 0.57, p_adj_ < 0.001) as well as to children (*M* = 0.78, *SD* = 0.67, *p*_adj_ = 0.020).

Given the heterogeneous patterns between the compared datasets, the question whether overarching interplays between pathogens can be detected seemed even more relevant. Accordingly, we proceeded to cluster analysis data for all pathogens displayed in Table 2.

The results of the cluster analysis were first stratified by dataset to analyze whether an overarching pattern could be detected across both datasets.

Dataset I

**Figure 1:**
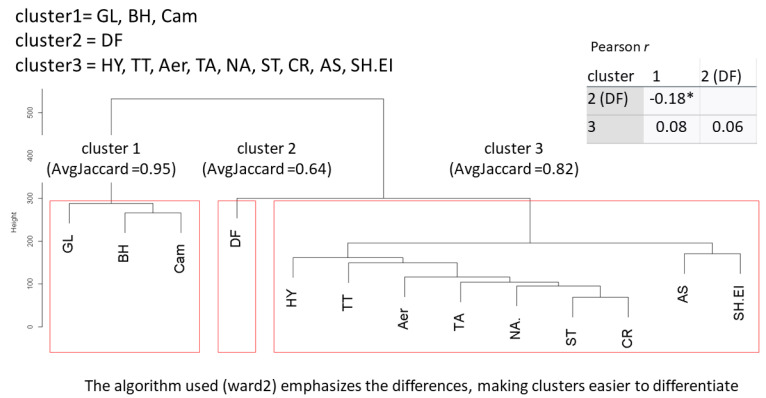
Cluster dendrogram for dataset I. GL = *Giardia lamblia*, BH = *Blastocystis hominis*, Cam = *Campylobacter* spp., DF = *Dientamoeba fragilis*, HY = *Hymenolepis nana*, TT = *Trichuris trichiura*, Aer = *Aeromonas* spp., TA = *Taenia* spp., NA = *Necator americanus*, ST = *Strongyloides stercoralis*, CR = *Cryptosporidium* spp., AS = *Ascaris lumbricoides*, SH.EI = *Shigella* spp./enteroinvasive *Eschericha coli* (EIEC). * = *p* < 0.05.

Information for all helminths, protozoa and bacteria were included in the analysis, screening in total for an interaction between 13 pathogens in dataset I. The Jaccard index indicates a stable to highly stable solution with a range of 0.65–0.95 for dataset I. *B. hominis*, *G. lamblia* and *Campylobacter* spp. form a distinct cluster. However, related to other parasites, *D. fragilis* demands a unique position, as do *A. lumbricoides* and SH.EI. Correlation between clusters is weak, in the range of r = −0.18 * −0.08, as was to be expected when using Ward’s minimum variance criterion. As indicated by the distance and height of the pathogens in the dendrogram, a small negative correlation occurs between *G. lamblia*, *B. hominis*, *Campylobacter* spp. and *D. fragilis* (*r* = −0.18, *p* < *0*.05) (see Figure 1). The same exploratory approach of k-means clustering was subsequently used to scrutinize pathogen interactions in dataset II.

Dataset II

Information for all helminths, protozoa and bacteria was included in the analysis, screening in total for an interaction between 13 pathogens in dataset II. The Jaccard index indicates a stable to highly stable solution ranging from 0.64 to 0.92.

**Figure 2:**
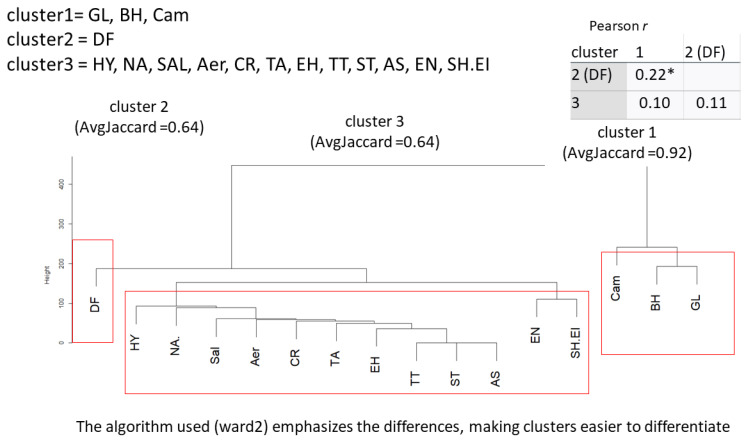
Cluster dendrogram for dataset II. GL = *Giardia lamblia*, BL = *Blastocystis hominis*, Cam. = *Campylobacter* spp., DF = *Dientamoeba fragilis*, HY = *Hymenolepis* nana, TT = *Trichuris trichiura*, Aer = *Aeromonas* spp., TA = *Taenia* spp., *N. americanus* = *Necator americanus*, ST = *Strongyloides stercoralis*, CR = *Cryptosporidium* spp., AS = *Ascaris lumbricoides*, SH.EI = *Shigella* spp./enteroinvasive *Eschericha coli* (EIEC). * = *p* < 0.05.

The results from dataset I were nearly replicated in independent dataset II (Figure 2), but the configuration is slightly different.

Again, *B. hominis*, *G. lamblia* and *Campylobacter* spp. form a distinct cluster with *D. fragilis*, demanding a unique position. *A. lumbricoides* and SH.EI. do not form a unique cluster but remain in a proximate configuration. Again, a weak correlation between cluster 1 and cluster 2 (*r* = 0.22, *p* < 0.05) is determined.

Since *B. hominis*, *G. lamblia*, *Campylobacter* spp. and *D. fragilis* were found in many samples and were characterized by a corresponding stable cluster solution, further analyses were focused on the interplay of these clusters. Structurally, they fuse hierarchically prior to contact with cluster 3, suggesting an exploratory moderated regression as an appropriate method to scrutinize their interplay.

Since the pattern of dataset I was verified by dataset II, further analysis was conducted with both datasets.

Exploratory moderated regression to scrutinize the interplay of the clusters

**Figure 3:**
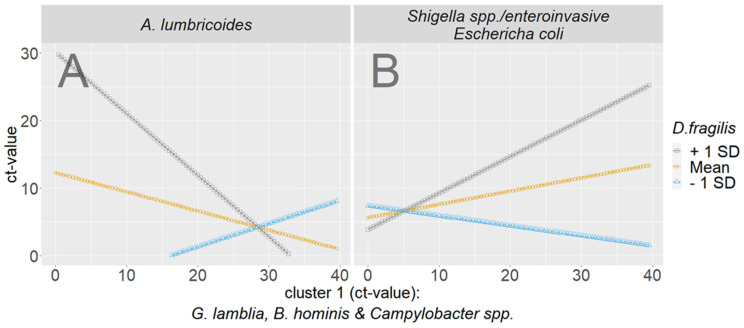
Moderated regression analysis with *A. lumbricoides* (**A**) and SH.EI (**B**) as the criteria.

Since the cluster analysis suggested that *D. fragilis* has a “gatekeeper” function for cluster 1 to interact with cluster 3, we aimed to investigate their interplay in a moderated regression. In the regression analysis, *B. hominis*, *G. lamblia* and *Campylobacter* spp. were employed as predictors and *D. fragilis* as a moderator to inspect their interaction on cluster 3. Since *A. lumbricoides* and SH.EI. are most present in cluster 3 and situated hierarchically lower in both cluster solutions, we focused on the prediction of these pathogens. Belonging to cluster 1 within both datasets, *B. hominis*, *G. lamblia* and *Campylobacter* spp. were inspected in combination. For *A. lumbricoides*, a significant moderated interaction occurred (Figure 3). While cluster 1 alone does not have significant predictive power (*b* = 0.60, *p* > 0.05), *D. fragilis* does (*b* = 1.15, *p* < 0.01), and so does the interaction between cluster 1 and *D. fragilis* (*b*= −0.04, *p* < 0.01). The slope becomes negative in the interaction.

In the case of *D. fragilis* detections with high CT-cycle threshold values (+1SD (standard deviation), low pathogen load, grey line), high CT values for *A. lumbricoides* are promoted (low pathogen load) solely if values of cluster 1 are low (high pathogen load). In other words, given a high pathogen load of *G. lamblia*, *B. hominis* and *Campylobacter* spp., in combination with a low pathogen load of *D. fragilis* (grey line), the pathogen load of *A. lumbricoides* decreases (high CT values). The opposite is true when there is a high pathogen load of *D. fragilis* (low CT values, blue line) in combination with a high pathogen load of *G. lamblia*, *B. hominis* and *Campylobacter* spp. (low CT values), whereby low-CT-value infection with *A. lumbricoides* becomes likely.

The analytically deduced moderation between cluster 1 (*G. lamblia*/*B. hominis*/*Camplylobacter* spp.) and SH.EI did not turn out to be significant (see Figure 3B). However, there seems to be a moderating tendency in the opposite direction compared to *A. lumbricoides*.

**Figure 4:**
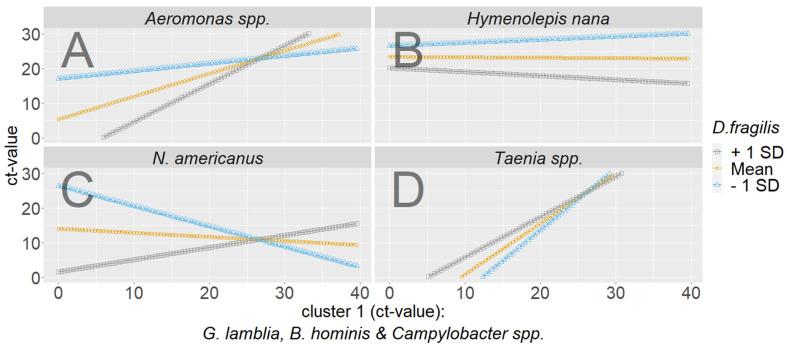
Interaction plots for *Aeromonas* spp. (**A**), *Hymenolepis nana* (**B**), *N. americanus* (**C**) and *Taenia* spp. (**D**) as criterion cluster 1: *B. hominis*/*Campylobacter* spp. *Giardia G. lamblia* as predictor and *D. fragilis* as moderator. HY = *Hymenolepis nana*, *N. Americanus* = *Necator americanus*, TA = *Taenia* spp., Aer = *Aeromonas* spp.

Moderation analysis was conducted for all other pathogens of cluster 3, for which at least five cases were reported. These were *H. nana*, *Taenia* spp., *N. americanus* and *Aeromonas* spp. The moderation by *D. fragilis* for these pathogens of cluster 3 was not significant, which needs to be interpreted carefully, since the calculations were based upon a relatively small sample size (Figure 4). The data may be of use as a starting point for investigating a complex interplay of multiple pathogens. In the case of *N. americanus*, low CT values of *G. lamblia*, *B. hominis* and *Campylobacter* spp. (high pathogen load) and low CT values of *D. fragilis* (high pathogen load/grey line) make an infection with *N. americanus* unlikely, but the tables turn if *D. fragilis* has high CT values (low pathogen load, blue line). In the case of *H. nana*, low CT values of *D. fragilis* (high pathogen load, blue line) seem to inhibit infection with HY, irrespectively of the CT values of *G. lamblia*, *B. hominis* and *Campylobacter* spp., whereas high CT values for *D. fragilis* (low pathogen load, grey line) make an infection with *H. nana* likely. For *Taenia* spp., a moderating interaction effect between *D. fragilis* and *G. lamblia* and *B. hominis* and *Campylobacter* cannot be assumed. The line runs almost parallel. For *Aeromonas* spp., low CT values of cluster 1 (high pathogen load) and high CT values of *D. fragilis* (low pathogen load, grey line) make an infection with *Aeromonas* spp. likely, whereas the opposite is true when CT values of *D. fragilis* are low (high pathogen load, blue line).

The study was conducted to retrospectively assess potential associations between various enteric pathogens of multiple infected or colonized Indigenous individuals in rural tropical Colombia. It its very rare to have such high infection rates within single individuals and populations; accordingly, there is only little information available about potential interactions between these pathogens.

The best modeling approaches were possible for *D. fragilis*, *B. hominis*, *Campylobacter* spp. and *G. lamblia*, because they occurred more frequently than other pathogens in the collected stool samples. The cluster analysis followed by moderated regression demonstrated a moderation between *B. hominis*, *Campylobacter* spp., *G. lamblia* and *D. fragilis* for *A. lumbricoides*.

In more detail, the CT-value-based associations showed a moderated regression between the *B. hominis*/*Campylobacter* spp./*G. lamblia* cluster, *D. fragilis* and *A. lumbricoides* depending on their quantitative abundance in the sample in a complex pattern. There was an antagonistic effect of *D. fragilis* towards cluster 1 consisting of *B. hominis*, *G. lamblia* and *Campylobacter* spp. The high abundance of pathogens of the *B. hominis*/*G. lamblia*/*Campylobacter* spp. cluster relies on a medium to low abundance of *D. fragilis* to support an infection with quantitatively high abundance of *A. lumbricoides*. There is clear evidence that *D. fragilis* moderates the impact of the *G. lamblia*, *B. hominis* and *Campylobacter* spp. cluster on infections with *A. lumbricoides*. There is further tentative evidence that *D. fragilis* acts as a moderator in combination with the *G. lamblia*, *B. hominis* and *Campylobacter* spp. cluster for the promotion or inhibition of the pathogen load of other pathogens of cluster 3 as well.

These results are insofar interesting, as they suggest an impact by the protozoa *B. hominis* and *D. fragilis*, the etiological relevance of which is quite controversially debated [14,15] with authors of case reports or small-sized cross-sectional studies occasionally claiming such relevance [16,17] in spite of conflicting study results [14]. The presented results, however, show that—depending on their concentrations—*D. fragilis* acts as a moderator, for example, for *B. hominis*/*Campylobacter* spp./*G. lamblia*. In certain amounts and constellations, microorganisms like *D. fragilis* can act as facilitators of infections with other pathogens (e.g., with *A. lumbricoides*), and, therefore, they might be noticed as facultatively pathogenic as well. 

Associations of *B. hominis* infection with the human microbiome in general [18,19,20,21], as well as with particular bacterial pathogen species like *Clostridioides difficile* [21,22,23], have been repeatedly suggested, and insofar, the observed association with a *Campylobacter* spp.-containing pathogen cluster is supporting this presumption. Associations of *B. hominis* detection and the co-detection of other parasites have been described [24,25,26], including different *B. hominis* lineages in the same patient [27] and even extra-intestinal human parasites like *Echinococcus* spp. [28], and the here-observed association of *B. hominis* and *G. lamblia* supports a previous report [29]. Last but not least, the reported higher *B. hominis* abundance in patients with acquired immunodeficiency should be mentioned as well [30]. In a similar way as described for *B. hominis*, *D. fragilis* has been repeatedly observed as a component of enteric co-infections [31,32]. Adding to such previous findings, their moderating ability is described in detail here, depending on their concentrations and co-infecting microorganisms.

Similar to what was observed in the assessment here, potential associations between *D. fragilis* and enteric helminth infections have been repeatedly discussed. In recent works, co-infections with *D. fragilis* and *Enterobius vermicularis* were primarily discussed as potentially etiologically linked [33,34]. In a Thai research report from the late 1970s, the abundance of *D. fragilis* in *A. lumbricoides* ova was claimed [35], a finding which might partially explain the here-described association between these two parasites. However, this potential interlink still does not explain the observed influence of the *B. hominis*/*Campylobacter* spp./*G. lamblia* cluster, again highlighting new potential linkages.

Although the sample size of the assessed datasets has been relatively small, the results are likely to be valid, as indicated by confirmation in a second dataset. Although more stool samples from Colombian Indigenous people had been diagnostically assessed for the different previous studies [1,2,3,4,5,6], only samples which had been analyzed with the same real-time PCR assays could be included to ensure comparability. Although the two different datasets showed quite similar results, the samples from both datasets came from Indigenous individuals living under comparable living conditions in the rural tropical north-east of Colombia. Another dataset from a completely different geographic region would have been desirable for comparison purposes. However, a respective broadening of the study design was not feasible due to funding constraints. Accordingly, it cannot be claimed with certainty whether the observed clusters are just a regional Colombian phenomenon or constitute a more general pattern. However, when checking for a systematic pattern of pathogens not present in the sample using Little’s MCAR (missing completely at random) test [36], no evidence was found that covariates such as food would have added significant information to the observed pathogen distribution. The latter gives statistical support that these findings are of a general character.

As an undeniable limitation, the applied commercial real-time PCR platform did not provide quantitative results. Insofar, the semi-quantification based on CT values as conducted in this study is only a measure of relative abundance of the target DNA within the analyzed samples. Further, and as typical for all diagnostic assays, the applied real-time PCRs cannot decide on the absolute abundance or absence of target DNA within the sample matrix, and technical detection thresholds have to be considered.

While modern approaches like capsule-based sample collection in the gut have been implemented to standardize enteric sampling [37], such sophisticated methodology was not available for infrastructural reasons in our study.

## 4. Conclusions

The provided assessment demonstrated a complex and quantity-dependent interplay between *B. hominis*/*Campylobacter* spp./*G. lamblia*, *D. fragilis* and *A. lumbricoides*, as well as stable pathogen clustering in Colombian Indigenous people living in resource-limited settings. Future studies are needed to confirm or deny the recorded patterns and to even more decipher the biological mechanisms of these proposed interactions, but can be added to the manuscript if the discussion is unusually long or complex.

## Figures and Tables

**Table 1:** Comparison of the datasets. For significant differences between datasets regarding average infections per person, maximum number of infections in one person and number of pathogens, the Welch test uses equal parameters. Therefore, the parameters of the Welch test are reported only once for “Average infections per person”.

Dataset				
*N* = 244	1 (Kogui) *n* = 150	2 (Wiwa) *n* = 94	Both	Differ Significantly?
Age *M* (*SD*)	23.35 (18.59)	21.01 (14.48)	22.45 (17.13)	No *t* (230.7) = 1.1, *p =* 0.27
Persons infected with at least one pathogen				
helminths *n* (%_column_)	35 (23%)	14 (15%)	49 (20%)	No *X*^2^ (1, *N =* 244) = 2.07, *p* = 0.15
protozoa *n* (%_column_)	138 (92%)	93 (99%)	231 (95%)	Yes *X*^2^ (1, *N* = 244) = 4.22, *p* = 0.04
bacteria *n* (%_column_)	83 (55%)	34 (36%)	117 (48%)	Yes *X*^2^ (1, *N* = 244) = 7.75, *p* = 0.005
Average infections per person (*M*, *SD*)				
helminths *n* (%_column_)	0.28 (0.56)	0.15 (0.36)	0.23 (0.49)	Yes *t* (241.83) = 2.24, *p* = 0.026
protozoa *n* (%_column_)	1.53 (0.73)	1.77 (0.72)	1.62 (0.74)	Yes *t* (198.7) = −2.50, *p* = 0.013
bacteria *n* (%_column_)	0.62 (0.62)	0.40 (0.57)	0.54 (0.61)	Yes *t* (209.07) = 2.77, *p* = 0.006
Maximum number of infections in one person				
helminths *n* (_occurrence_)	3.00 (1)	1.00 (14)	3.00 (1)	
protozoa *n* (_occurrence_)	3.00 (9)	4.00 (1) [3 pathogens found 12 times]	4.00 (1)	
bacteria *n* (_occurrence_)	3.00 (1)	2.00 (4)	3.00 (1)	
Number of pathogens				
helminths *n* (%_column_)	42 (28%)	14 (15%)	54 (22%)	
protozoa *n* (%_column_)	229 (152%)	166 (176%)	395 (161%)	
bacteria *n* (%_column_)	93 (62%)	38 (40%)	131 (54%)	

**Table 2:** Occurrence of pathogens within the compared datasets.

		1 (Kogui) *n* = 150	2 (Wiwa) *n* = 94
Microsporidia	*Encephalitozoon* spp.	0	7
Helminths	*Strongyloides* spp.	2	0
	*Hymenolepis* spp.	11	3
	*Ascaris* spp.	12	0
	*Taenia* spp.	4	1
	*Trichuris trichiura*	9	0
	*Necator americanus*	4	3
Protozoa	*Blastocystis hominis*	132	90
	*Giardia lamblia*	55	59
	*Dientamoeba fragilis*	41	15
	*Entamoeba histolytica*	0	1
	*Cryptosporidium* spp.	1	1
Bacteria	*Shigella* spp./EIEC	11	7
	*Campylobacter* spp.	78	29
	*Aeromonas* spp.	4	1
	*Salmonella* spp.	0	1

## Data Availability

All relevant data are provided in the manuscript. Raw data can be made available at reasonable request.
